# Health coach-supported mobile health intervention to improve adherence to lipid-lowering medications (AdLip): Design and rationale of a multicentre randomized controlled trial protocol

**DOI:** 10.1371/journal.pone.0346509

**Published:** 2026-05-05

**Authors:** Qi Chwen Ong, Emina Obarcanin, Iva Bojic, Sock Hwee Tan, Sameera Senanayake, Sanjeewa Kularatna, Nicholas Graves, Siew Pang Chan, Jun Long Marvin Sim, Derek Hausenloy, Mark Yan Yee Chan, Andy Wai Hoong Khong, Doreen Su-Yin Tan, Andy Hau Yan Ho

**Affiliations:** 1 Lee Kong Chian School of Medicine, Nanyang Technological University, Singapore, Singapore; 2 Cardiovascular Disease National Collaborative Enterprise, Consortium for Clinical Research and Innovation, Singapore, Singapore; 3 Health Services & Systems Research, Duke-National University of Singapore Medical School, Singapore, Singapore; 4 National Heart Research Institute Singapore, National Heart Centre, Singapore, Singapore; 5 Cardiovascular Research Institute, National University Health System, Singapore, Singapore; 6 Centre for Behavioural & Implementation Science Interventions, Yong Loo Lin School of Medicine, National University Singapore, Singapore, Singapore; 7 Institute of Geriatrics & Active Ageing, Tan Tock Seng Hospital, Singapore, Singapore; 8 National University Health Systems Pharmacy, Singapore, Singapore; 9 Cardiovascular & Metabolic Disorders Program, Duke-National University of Singapore Medical School, Singapore, Singapore; 10 The Hatter Cardiovascular Institute, University College London, London, England, United Kingdom; 11 Department of Cardiology, National University Heart Centre, National University Hospital, Singapore, Singapore; 12 School of Electronic and Electrical Engineering, Nanyang Technological University, Singapore, Singapore; 13 Department of Pharmacy and Pharmaceutical Sciences, National University of Singapore, Singapore, Singapore; 14 School of Social Sciences, Nanyang Technological University, Singapore, Singapore; 15 Palliative Care Centre for Excellence in Research and Education, Singapore, Singapore; PLOS: Public Library of Science, UNITED KINGDOM OF GREAT BRITAIN AND NORTHERN IRELAND

## Abstract

**Background:**

Elevated low-density lipoprotein cholesterol is one of the major modifiable risk factors for cardiovascular disease. Lipid management is integral to primary and secondary prevention of cardiovascular disease, but undermined by high prevalence of non-adherence to lipid-lowering medications. Innovative, personalized multicomponent interventions may address the gaps in medication non-adherence. We aim to evaluate the effectiveness of a human coach-supported digital personal health assistant (mobile health app) intervention in improving adherence to statins in adults with hyperlipidaemia.

**Methods:**

AdLip is a multicentre, open-label, parallel, two-arms, randomized controlled trial aiming to recruit a minimum of 376 adult participants who are non-adherent to statins from primary care ambulatory clinic setting in Singapore. Participants recruited will be randomly assigned (1:1) to human-coach supported mobile health app intervention or control group. The primary outcome will be adherence to statins at 6 months, measured by Medication Adherence Report Scale-5. Secondary outcomes will include change in serum low-density lipoprotein cholesterol levels, health motivation and attitudes, self-efficacy, self-care behaviours, quality of life, app acceptability, user engagement, and cost-effectiveness. Outcomes will be analysed using intention-to-treat approach.

**Discussion:**

The AdLip trial will provide empirical evidence on a multicomponent approach to the long-standing challenge of suboptimal adherence to statins in a multi-ethnic Asian setting. Findings from this study may inform a more personalized approach to addressing non-adherence in the short term, and how it relates to cardiovascular disease prevention in the longer term.

**Trial Registration:** ClinicalTrials.gov NCT06614049.

## Introduction

Elevated low-density lipoprotein (LDL) cholesterol remains one of the leading modifiable risk factors for cardiovascular disease (CVD) [1]. Since 1990, the global CVD burden attributable to high LDL cholesterol has risen steadily, contributing significantly to increases in disability-adjusted life years, years lived with disability, and years of life lost [1]. In Singapore, the incidence of acute myocardial infarction in patients with hyperlipidemia is projected to rise by 205% between 2025 and 2050, reaching 1041 per 100,000 population [2]. Lipid management is integral to the prevention of CVD [3]. Statins can significantly reduce the risk of CVD events and mortality in both primary and secondary prevention populations [4,5]. Although statins are highly efficacious and widely prescribed for lipid management [6,7], non-adherence to statins remains prevalent worldwide [8]. Approximately 60% of patients stopped taking statins within six months of initiation [9], and up to 75% discontinued statins by the end of first year [10]. This is concerning as statin non-adherence is associated with substantially increased risk of CVD and mortality [11].

Medication non-adherence is multifactorial. It can be influenced by a range of factors related to patients, their medical conditions, treatment regimens, the healthcare system, or socioecomonic circumstances [12]. Among patient-related factors, intentional factors are linked to beliefs or attitudes about the condition or medications, exposure to misinformation, expectations for improvement, and deliberate discontinuation [13,14]. In contrast, unintentional non-adherence may result from forgetfulness, poor understanding of medication schedules and dosages, low health literacy, and logistical barriers such as medication cost and transportation challenges [15,16]. For statins non-adherence, nocebo effect is a unique factor commonly reported as reason for discontinuation [17]. Multiple strategies can be used to improve adherence to medication. These include patient education, medication reminders, simplifying medication regimens, and providing incentives for adherence [18–20]. However, addressing medication non-adherence is challenging. Various contributing factors are likely to influence one another, thereby limiting the effectiveness of single-factor interventions [21]. The complex nature of medication non-adherence suggests that a multicomponent intervention is often required [22].

Mobile health apps (mHealth) are proven to be effective for chronic disease management and cardiac rehabilitation [23,24]. These technologies significantly improved medication adherence and health outcomes in patients with chronic diseases [25,26]. However, the improvements in medication adherence were difficult to sustain [27]. Additionally, user engagement is a key challenge that limits the effectiveness of mHealth interventions [28]. Health coaching has the potential to promote sustained behaviour change and enhance user engagement with mHealth apps. Through a person-centred and individualized approach, health coaches leverage a range of behavioural theories and principles to support individuals in achieving their health-related goals and improving overall well-being [29]. A systematic review showed that health coaching had positive effects on physiological, behavioral and psychological aspects of patients with chronic diseases [30].

Given the shared focus on personalised behavioural interventions in both mHealth and health coaching, their integration may offer synergistic benefits. This is supported by a scoping review indicating that mHealth and health coaching interventions could potentiate each other [31]. Although previous pilot trials demonstrated positive impact of combined mHealth and health coaching intervention on cardiac rehabilitation and chronic low back pain [32,33], its effect on medication adherence is not known. Evidence from randomized controlled trials on the effectiveness of this multicomponent intervention is also scarce. In the AdLip trial, we aim to investigate whether a human coach-supproted mobile health app intervention improves adherence to statin therapy in adults with hyperlipidaemia.

We hypothesize that the use of a health coach-supported mobile health app intervention will improve adherence to lipid-lowering medications in adults with hyperlipidaemia. The primary objective is to evaluate the effectiveness of a human coach-supported mobile health app intervention on adherence to lipid-lowering medication in adults with hyperlipidaemia at 6 months when compared to usual care. Secondary objectives include evaluating the effects on serum LDL cholesterol, health motivation and attitudes, self-efficacy, self-care behaviours, quality of life, usability of the app, and cost-effectiveness of the intervention.

## Materials and methods

### Study design

**Fig 1:**
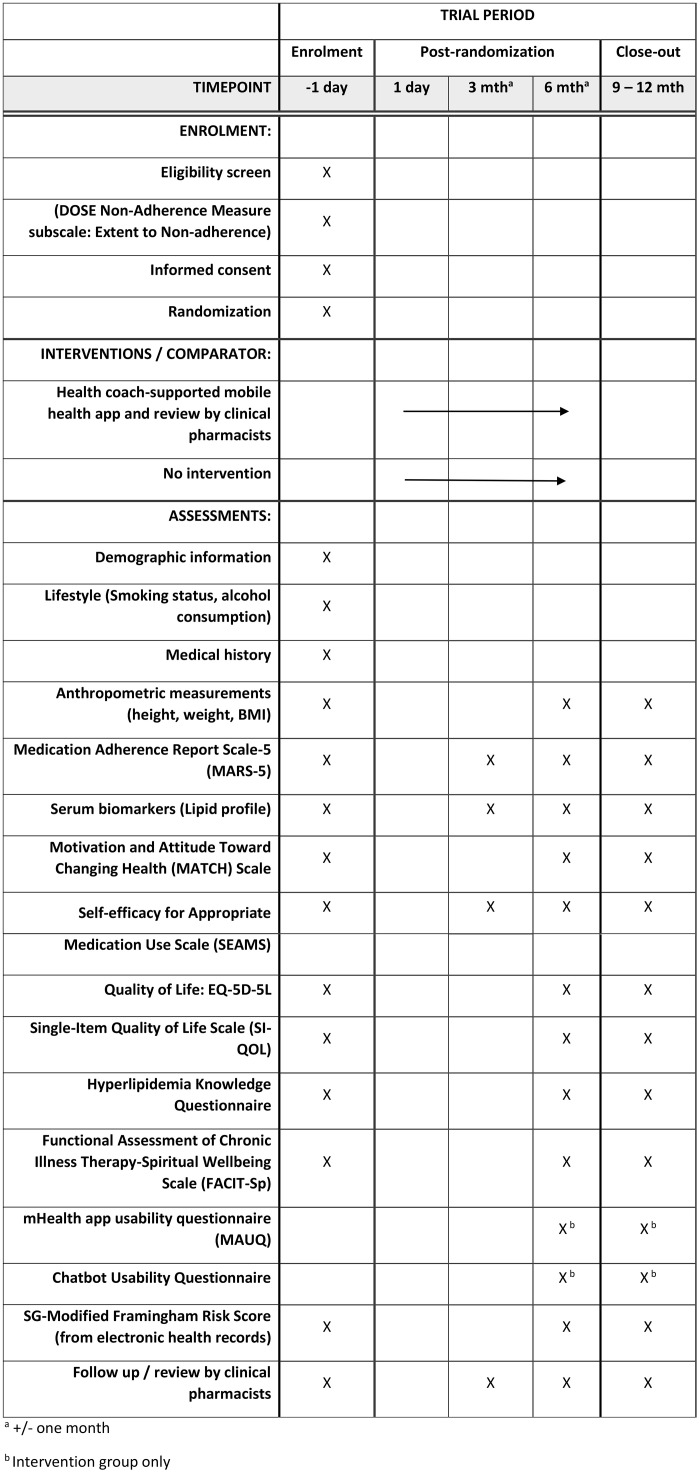
The schedule of enrolment, interventions, and assessments.

AdLip is a multicentre, open-label, parallel, two-armed randomized controlled trial (RCT) comparing the use of health coach-supported mobile health app for adherence to lipid-lowering medications in adults with elevated LDL cholesterol compared with usual care at 6 months. The schedule of enrolment, interventions, and assessments can be seen in Fig 1. This protocol was prepared in accordance with the SPIRIT (Standard Protocol Items: Recommendations for Interventional Trials) Statement (S1 File) [34].

### Participants

#### Inclusion criteria.

The trial will recruit participants from primary care ambulatory clinic setting who meet the following criteria: (1) Aged between 21–84 years old; (2) Prescribed or intent to restart statins with or without ezetimibe for lipid management; (3) Medication non-adherence as defined by the “Extent of Non-adherence” sub-scale of the DOSE Non-Adherence Measure, with a score ≥ 1 (range from 0–3); (4) Singapore residents (citizens, permanent residents, or long-term pass holders); (5) In possession of a smartphone or tablet with Android or iOS operating systems; (6) Have internet access on their mobile devices.

#### Exclusion criteria.

Participants will be excluded if they meet any of the following: (1) Does not read or understand English; (2) Current use of smartphone medication adherence app(s) that include statins; (3) Concurrent use of PCSK9 inhibitors in addition to statins and/or ezetimibe; (4) Participation in another study that uses medications that could affect lipid levels; (5) Severe renal impairment defined as chronic kidney disease stage 4 and above; (6) Severe liver disease (e.g., Child-Pugh Class C); (7) Existing musculoskeletal-related complaints or diagnoses which may confound adverse event reporting; (8) Uncorrected thyroid conditions, especially poorly-controlled hypothyroidism; (9) Documented psychiatric diagnosis, history of mental illness, or deemed as unable to give informed consent; (10) Currently pregnant, breastfeeding, or expecting to get pregnant during the course of the study; (11) Guarded prognosis with expectant mortality within 12 months or less.

### Study setting and recruitment

Trial participants will be recruited from multiple primary care ambulatory clinics in Singapore by clinical research coordinators (CRC). Participants will be identified through electronic database pre-screening or referrals from the primary care team (PCT), including physicians, nurses, program managers, or pharmacists. If a patient expresses willingness to consider the study, their PCT will refer them to a CRC. The CRC will then approach the referred patients, screen for non-adherence using the DOSE questionnaire, and assess their eligibility based on the study’s inclusion and exclusion criteria. Eligible patients will receive verbal explanation of the study and a participant information sheet from the CRC. Informed consent will be obtained from participants in the presence of an independent witness.

### Randomization, allocation concealment, and blinding

Enrolled participants who have completed the baseline assessment will be randomly assigned to the intervention group or the control group at a 1:1 ratio. A trial statistician who is not involved in the recruitment process will use a pre-defined study ID list to perform block randomization with random permuted block sizes of 4 and 6, using R statistical software package. Study allocation based on assigned Study ID will only be made known to the attending CRC and the patients after eligibility and informed consent are sought. Since the study mobile app will only be allocated to the intervention group, an open-label design is used and blinding of participants, PCT, CRCs, and study investigators is not possible.

### Control

Participants in the control group will receive usual care consisting of scheduled consultations with their PCT at their respective primary care ambulatory clinic. They may also receive other forms of support during the consultations or other dedicated times, subject to their PCT’s clinical judgement. Licensed collaborative prescribing practitioners (i.e., clinical pharmacists) will follow up with participants to titrate lipid-lowering medications against their serum lipid levels. Blood creatinine kinase and liver function tests are repeated when indicated.

### Intervention

In addition to usual care, the intervention group will receive a 6-month multicomponent intervention delivered via a digital platform consisting of mobile health app, digital health coaching portal, and administration portal.

#### Health coach-supported mobile health app.

The CADENCE digital personal health assistant (D-PHA) is a multi-component mobile health platform integrated with virtual health coaching. Developed by the study team, the D-PHA platform was designed to improve adherence to medications and lifestyle modifications through health education, improving their medication-taking behaviors, and adopting more healthy lifestyle behaviors. The behavioral change driven by the app was designed based on the established behaviour change technique taxonomy (v1) of 93 techniques hierarchically organized into 16 higher-order categories, including goals and planning, feedback and monitoring, social support, shaping knowledge, and self-belief [35]. In addition, two newly proposed categories used in digital therapeutic interventions, which are personalization and gamification, were also incorporated. To augment participants’ lifestyle behaviours and promote adherence to medication, the app consists key features such as educational modules, medication reminders, conversational agent (rules-based chatbot), and in-app communication with health coaches.

Educational modules cover content related to medication therapy for lipid management and the sequelae of poor lipid management, developed by a multidisciplinary team of medical doctor, pharmacist, and health psychologist. The content was structured based on Health Belief Model and Transtheoretical Model of Change [36,37]. To ensure clinical accuracy and contextual relevance, an expert review panel consisting of primary care physicians, cardiologist, pharmacists, dietitian, health psychologist, and patient advocate was convened to review the content, which covers six themes: (1) Introduction to heart health; (2) Lipids and Cardiovascular Disease; (3) Lipid-loweing medications; (4) Importance of medication adherence; (5) Heart-healthy behaviours and mindsets; and (6) Towards sustainable heart-healthy living. Each theme is made up of several modules, which comprise multiple units. Each unit is made up of a video recording or animation accompanied with transcripts, followed by a poll question or knowledge quiz to help participants reflect their understanding of the content.

Reminders and logs features are available on the D-PHA app, allowing participants to record food diaries and receive reminders for medications and clinic appointments. The platform also sends reminders via in-app text messages and push notifications to promote app usage and medication adherence.

Rule-based chatbot was designed to engage participants in conversations that uncover their perceived barriers to medication adherence and assists them with setting their personal health goals. Their interactions with the chatbot are tracked and made available to health coach to inform personalized coaching.

#### Health coaching.

The health coaching component of the intervention is delivered digitally via the D-PHA app. Health coaches are given access to a designated dashboard where they can schedule health coaching sessions, review app usage, and interacts with their assigned participants through asynchronous (text messaging) and synchronous communication (video-call). A special focus will be devoted to medication adherence through gathering information on participants’ knowledge regarding medication-taking behaviours and associated side effects, as well as identifying discrepancies between the list of prescribed lipid-lowering medications and participants’ self-reported use of the medications.

The sessions are delivered by health coaches who underwent two training programs. They completed a core coach training and certification program approved by the National Board for Health & Wellness Coaching (NBHWC) [38]. Additionally, they participated in a focused program on health coaching for older adults, offered by a higher education institution in Singapore [39]. This dual training approach ensures that health coaches acquire core coaching competencies and are capable of applying them effectively within the local context.

Although there is a lack of consensus on the intensity and frequency of coaching sessions, existing literature suggests the duration of effective health coaching programs to be between 6–12 months to achieve behaviour change, with 6 months being reported most frequently [30,40]. The number of sessions ranged from 1 to 12 [41]. Based on these findings, the minimum number of coaching sessions between participants and health coaches is set at four, with each session taking place once a month and lasting up to 60 minutes. After completion of four health coaching sessions, participants may request for two additional sessions if needed. Participants are encouraged to text their health coaches via the D-PHA app for additional support in between health coaching sessions. Health coaches may also initiate additional interactions with the participant if the app’s usage analytics suggest low engagement.

During the session, health coaches will follow a pre-specified health coaching guidelines customized for the trial to minimize discrepancies among health coaches. They will review adherence to prescription instructions, explore reasons for non-adherence, assist participants in setting SMART (Specific, Measurable, Achievable, Realistic, and Time-bound) behavior goals, and review goals and app usage. Health coaches will not provide any medical advice or instruction, including starting, stopping, or titrating medications. The scope of health coaching will be strictly related to medication adherence, lifestyle behaviours, and general health and well-being. Health coaches will keep a log detailing the communication with the participant.

#### Usage analytics for health coaching.

These information provide insights into participants’ engagement with the various features of the app. Passively collected data such as the number of logins, modules completion, time spent online, and other click-level data are captured and analysed. These data are presented on the dashboard of a central management system (i.e., administration portal). Additionally, each health coach has access to a designated dashboard to arrange coaching session, chat with patients, and monitor their app usage.

Before each session, health coaches will review the information gathered from usage analytics to guide and plan the upcoming session. The information is displayed on the health coach dashboard and includes medication logs, food diary entries, learning progress in the educational component, interactions with chatbot, and reflection journal entries. A weekly action score summarizing participant’s weekly activity in the app will also be shown to health coach. Information collected by the rule-based chatbot, such as the user’s readiness to change, perceived barriers and misbeliefs may also be used to guide a targeted behavioural intervention.

### Outcomes

The primary outcome is the improvement in medication adherence to lipid-lowering medications, measured using Medication Adherence Report Scale-5 (MARS-5) at 6 months. Secondary outcomes include (1) Change in serum LDL cholesterol levels; (2) Change in Singapore-modified Framingham Risk Score 2023 (SG-FRS-2023) [42]; (3) Change in health motivation assessed by the Motivation and Attitude Toward Changing Health (MATCH) Scale [43]; (4) Change in self-efficacy assessed by Self-Efficacy for Appropriate Medication Use Scale (SEAMS); (5) Change in self-reported quality of life assessed by the EQ-5D-5L [44], the Single-Item Quality of Life Scale (SI-QOL) [45], and a concise version of the Functional Assessment of Chronic Illness Therapy – Spiritual Well-Being Scale (FACIT-Sp) [46]; (7) App usability assessed by mHealth app usability questionnaire (MAUQ) [47]; (8) Chatbot usability assessed by Chatbot Usability Questionnaire at 6 months; (9) Adherence to the intervention (health coaching attendance and app usage); and (10) Cost-effectiveness of the intervention through quality adjusted life years (QALYs) gained and incremental cost effectiveness ratios (ICER): cost per QALY gained.

### Cost-effectiveness analysis

#### Economic evaluation.

A cost-effectiveness study will be conducted to investigate the expected changes to total costs and health benefits, as measured by Quality Adjusted Life Years, from a decision to adopt the human coach-supported digital personal health assistant. A Markov model, to achieve this, will be defined by the natural history of a patient with hyperlipidemia. The analysis will consider only the health system perspective. The structure of the model will be validated with cincial experts. The following information is needed: transition probabilities, costs, and effectiveness measures.

The transition probabilities will be sourced from the data collected in the trial (e.g., risk categories according to the Singapore-modified Framingham Risk Score 2023, adherence rate), and from the literature.

Intervention costs will be estimated using the information available within the trial budget. The cost of developing the app as a one-off cost, training the personnel, and maintenance cost will be calculated. Furthermore, the costs of statin treatment and the costs of cardiovascular events will be estimated and will be used as model inputs. Cost-effectiveness, healthcare utilisation, and direct medical costs will be collected.

The study will use several effectiveness measures. For health economic evaluations, effectiveness measures must be either in natural measurements, such as hospital avoidance, or patient-reported outcome measures, such as utility estimates. The effectiveness measures that will be used are EQ-5D-5L utility estimates (quality-adjusted life years – QALY), the number of hospital admissions, ED presentations and unplanned primary care visits avoided.

#### Model evaluation.

During analysis, statistical distributions will be used to describe uncertainty in model parameters. The normal, uniform, beta, and gamma distributions will be employed depending on the type of parameter. Fitted distributions will be randomly re-sampled and the economic outcomes of ‘change to total costs’ (ΔC) and ‘change to total effectiveness’ (ΔE) will be simulated 10,000 times. This approach propagates uncertainty in prior parameters forward to the total cost and outcomes. A decision rule for cost-effectiveness is given by (ΔC/ ΔE) <λ, where λ is the decision maker’s maximum willingness to pay (WTP) for health benefits. In the absence of a WTP value for Singapore, for this study, we use a value equalivent to three times that of the per capita GDP in Singapore. The flexibility of a Markov model will be used, with different scenarios being analysed to produce meaningful results for different stakeholders.

### Study procedures

#### Baseline.

Participants in both control and intervention groups will be asked to complete a series of self-reported questionnaires. Lipid profile, anthropometric measurements, and Singapore-modified Framingham risk score will be obtained from electronic health records. Participants assigned to the intervention group will be assisted by the CRCs to install the D-PHA app into their mobile devices and register a user account using a pre-specified User ID. The CRC will introduce different features of the D-PHA app through an “on-boarding” encounter.

#### Follow-up Visits.

The study has three follow-up visits at month 3, month 6, and month 12. Attending PCT will plan the follow-up visits to coincide with their regular clinic visits. Before each follow-up visit, CRCs will send reminders to participants. Data on clinician-reported outcome measures and patient-reported outcome measures will be obtained during each follow-up visit (Fig 1). Additionally, participants in the intervention group will complete the mHealth app usability questionnaire (MAUQ) and Chatbot Usability Questionnaire at month 6 and month 12.

### Sample size

Taking into account (1) a desired 5% level of significance; (2) a desired statistical power of 90%; (3) the potential application of a mixed-effect model given the multi-centre design, and to cater for the random effects of repeated measurements; (4) a small effect size to be detected (i.e., < 0.2); (5) fewer than 10 predictors to be accommodated in model building; and (6) no more than 20% dropouts, a total of 376 subjects (188 per arm) are to be recruited. However, based on previous experience with digital health based studies and numerous timepoints for patient-reported outcomes measures, the dropouts are anticipated to be larger than 20%. As such, a total of 450 subjects are to be recruited. A post-hoc power calculation will be facilitated after data analysis is performed.

### Statistical methods

The data collected will be screened for accuracy, missing data, outliers, and statistical assumptions. Following the principles of intention-to-treat (ITT) analysis, the recruited subjects will be retained in the group they are randomized to. At each measurement time point, efforts will be made to minimize missing data, and the reasons for loss of follow-up will be recorded. Missing data will be imputed using averages of answered items with no more than 20% missing data. When more than 20% of the items are missing, the overall score is not computed, and the data are considered missing. Sensitivity analyses will also be performed to determine the robustness of the assumptions about missing data.

Descriptive statistics are reported with mean ± standard deviation, median (interquartile range), and proportions (percentage). Exploratory analyses are performed with Pearson χ2 or Fisher’s exact test, independent t-test and Mann–Whitney U test, depending on the nature of data.

The default three-level random-intercept model is considered, in view of the multi-centre and longitudinal features of the study. That is, the repeated measreuments are nested within subjects, who are then nested within their respective centres. A random-slope model may be considered it it offers a better fit, and with justifiable clinical inisghts. The specific underlying distributions and link functions are chosen according to the nature of primary and secondary outcomes under investigation. The intraclass correlation is routinely reported for ascertaining the model fit. The adjusting variables include gender, disease duration, number of comorbidities, and lipid-lowering agents (statins only, or ezetimibe, or statins plus ezetimibe). Subgroup analysis may be performed if the finite mixture model (FMM) covers undelying sub-populations within the sample. An interim analysis may be performed depending on the situation. Analyzed with STATA MP V19 (Stata Corp,Texas, USA), R Studio (The R Foundation for Statistical Computing; Vienna, Austria), all statistical tests are conducted at 5% significance level or with its equivalence (i.e., 95% confidence intervals).

### Trial registration, human research ethics, dissemination plan, and data management plan

The trial received approval from the Domain Specific Review Board of the National Healthcare Group (**NHG DSRB 2023/00438**) on 29 Dec 2023 and was registered at ClinicalTrials.gov (**NCT06614049**). The main trial results will be published in the name of the AdLip Investigators. The authors are solely responsible for the design and conduct of this study, all study analyses, drafting and editing of the paper, and its final contents. Publication authors must meet the International Committee of Medical Journal Editors guidelines for authorship. Findings from this study will be presented at national and international meetings and published in peer-reviewed journals. Data will be made available through data access agreements established following approval through the CADENCE Governance Committee. Trial data will not be publicly released or placed into an open-access repository.

Informed consent will be sought from all participants by CRCs at study sites and/or study investigators. Written informed consent will be sought: 1) after the eligibility screening and participants’ verbal agreement, and 2) before baseline assessment and randomisation. Eligible participants will be given adequate time to decide whether they want to participate in the study. They will be encouraged to raise any questions to the CRC and to discuss their decision with family members. Ethical principles from the Declaration of Helsinki and Good Clinical Practices will be followed. If participants would like to withdraw from the study, they will be able to do so at any point without any implication of the medical support from the clinics. In the case of withdrawal, they are required to inform the CRC or study investigators, and no new data will be collected subsequently. Data that has been collected until the time of withdrawal will, however, be kept and used in the study. Any protocol amendments will be submitted to the Domain Specific Review Board of the National Healthcare Group for review and approval. Upon approval, the CRCs will inform participants of the changes during their next clinic visit.

All research data will be securely stored. Completed questionnaires and informed consent forms will be kept in locked cabinets at the respective recruitment sites, while electronic data will be password-protected and stored on secure institutional servers. Only authorized study team members, including the principal investigator (PI), co-investigators (CO-I), clinical research coordinators, and pharmacists will have access to the research data. Furthermore, each participant will be identified only by a screening or patient ID number. No data will be disclosed to any third party without participant consent, except when required for monitoring, auditing, or inspection by regulatory authorities or ethics committees. These are done to ensure that all study information remains safe and confidential.

### Safety management plan

Risk of harm is expected to be minimal from participation in the study. Safety and wellbeing will be assessed continuously in all patients participating in the study throughout the trial period. PI and CO-I will provide day-to-day oversight of the trial. They will assure that informed consent is obtained prior to performing any research procedures, that all patients meet eligibility criteria and that the study is conducted according to the approved research plan.

PI will evaluate the progress of the research study, including recruitment and retention, protocol deviations and an assessment of the timeliness and quality of the data on a biannual basis. PI will also review the collected data including adverse events and serious adverse events, unanticipated problems and withdrawal to determine if there is any change to the anticipated benefit-to-risk assessment of study participation. PI will ensure all protocol deviations, adverse events and serious adverse events are reported according to the applicable regulatory requirements.

If a participant sustains a physical injury as a result of study procedures performed in accordance with the approved protocol by the study doctors, the medical expenses required for the treatment of that injury will be compensated. Management of the normally expected consequences of standard medical treatment will not be covered. This compensation will be provided without legal commitment and without requiring participants to establish fault. There are conditions and limitations to the extent of compensation provided and particpants may discuss with the PI for more details.

### Trial status

Recruitment for the trial began in June 2024 and is expected to continue until June 2026. Recruitment sites were launched in phases, and at the time of manuscript submission, four sites were active: Jurong Polyclinic, Bukit Batok Polyclinic, Choa Chu Kang Polyclinic, and the National University Heart Centre.

## Discussion

Elevated LDL cholesterol is a major contributor of global CVD burden and continues to rise in prevalence worldwide [1]. Despite the high efficacy and wide availability, adherence to lipid-lowering medications remains poor [8]. Non-adherence to medications stems from a multitude of factors related to patients, their medical conditions, healthcare system, and socioeconomic circumstances, and often requires multifaceted strategies to address effectively [12,22]. The AdLip trial aims to investigate whether a health coach-supported mobile health app intervention can improve adherence to lipid-lowering medications in adults with hyperlipidemia. Results from this study could provide empirical evidence on the effectiveness of app-delivered health coaching interventions for chronic disease management in a multi-ethnic and multicultural Asian context. Furthermore, the trial’s focus on prevention of CVD aligns strategically with Healthier SG, a national initiative by Singapore’s Ministry of Health that emphasizes preventive healthcare [48].

Digital health interventions, particularly mHealth, have been studied extensively for their impact on medication adherence in chronic diseases. A meta-analysis reported that while mHealth, telehealth, and mHealth plus telehealth interventions were generally effective in lowering systolic and diastolic blood pressure, significant improvements in medication adherence were observed only in the mHealth group [49]. The suggestive effectiveness of mHealth interventions was consistent with findings from other systematic reviews examining mHealth interventions for medication adherence in diabetes and CVD [26,50]. Despite these promising outcomes, high percentage of users with minimal app engagement and high attrition rates remain substantial challenges in mHealth trials [51,52]. Research indicates that integrating health care professionals support into the mHealth apps could enhance user engagement [53]. This suggestion is echoed by a scoping review, which highlights the complementary role of health coaching alongside mHealth interventions [31].

Although a previous randomized controlled trial investigated an mHealth app paired with virtual health coaching intervention for medication adherence in cardiac rehabilitation, it was terminated early due to difficulties in enrolment (e.g., app compatible with iOS devices only), collection of primary outcome data, and withdrawal of mHealth app provider [54]. In the AdLip trial, MARS-5 will be used as the primary outcome measure instead of proportion of days covered to overcome practical challenges with pill count ascertainment, such as patients not bringing their pill bottles [54]. This, however, introduces the limitation of relying on a subjective measurement of medication adherence. The D-PHA app developed for the AdLip trial offers greater technological compatibility, with pre-release testing indicating its compatibility with most iOS and Android devices. Moreover, the D-PHA app was co-developed with an experienced vendor and is fully owned by the academic institution, reducing vulnerability to technology supplier withdrawal.

The AdLip trial nonetheless faces several potential limitations and challenges. Firstly, the app content and health coaching will only be available in English, thereby precluding those without English proficiency and limiting inclusivity of the trial. Secondly, although mobile phone ownership is used as an inclusion criterion, it may not accurately reflect participant’s digital literacy, which could influence their engagement with the app. Thirdly, the trial may be subject to contamination. This is because participants who attend the same polyclinics may live in the same community and interact with each other outside the study setting. High dropout rates are also anticipated, as this was reported as a major challenge in app-based RCTs on chronic diseases [52]. However, the use of asynchronous messages and contact with health coaches in this study may help improve retention and sustain longer-term engagement. Lastly, as the app was customized for the AdLip trial, the findings may not be directly generalizable to other app-based interventions.

In conclusion, the AdLip trial represents an important initiative towards addressing the challenge of medication non-adherence among patients with hyperlipidemia in Singapore. By integrating mobile health technologies with health coaching, this innovative approach offers opportunities to promote personalized, patient-centred care and enable task-shifting from primary care physicians to allied health professionals. If proven effective, this intervention may be expanded to the management of other non-communicable diseases, such as diabetes mellitus and hypertension.

## Supporting information

S1 File SPIRIT checklist. Recommended items to address in a clinical trial protocol and related documents.
(DOCX)

S2 File Protocol approved by the ethics committee. The original protocol approved by The Regional Committee for Medical and Health Research Ethics.
(DOCX)
