# Supplementary material for: Health coach-supported mobile health intervention to improve adherence to lipid-lowering medications (AdLip): Design and rationale of a multicentre randomized controlled trial protocol
Source: PLoS One. 2026 May 5;21(5):e0346509. doi: 10.1371/journal.pone.0346509 (PMC13143087; doi:10.1371/journal.pone.0346509)
Supplement: S2 File — The original protocol approved by The Regional Committee for Medical and Health Research Ethics. (DOCX) [file pone.0346509.s002.docx]

| **OFFICIAL USE ONLY** | |
| --- | --- |
| **Doc Name :** Protocol Template | |
| **Doc Number :** 207-002 | |
| **Doc Version :** 03 | **Date :** 01 June 09 |

**STUDY PROTOCOL**

| **PROTOCOL TITLE:** | |
| --- | --- |
| **AdLip:** Use of Human Coach-supported Digital/AI Personal Health Assistant to Improve Adherence to Lipid-Lowering Medications: A multi-centre randomised controlled trial | |
|  | |
| **PROTOCOL NUMBER:** | |
|  | |
|  | |
| **PROTOCOL VERSION:** | 1.0 |
| **PROTOCOL DATE:** | 20 Nov 2023 |
|  | |
| **PRINCIPAL INVESTIGATOR:** | |
|  | |
| **NUH:** Mark Chan Yan Yee | |
| **NUP:** Chua Ying Xian | |
| **NHGP:** Lee Eng Seng | |
|  | |
| **Recruitment SITE:** | |
| National University Polyclinics | |
| National Healthcare Group Polyclinics. | |
|  | |
|  | |
|  | |
|  | |
|  | |
|  | |

**TABLE OF CONTENTS**

[1. BACKGROUND AND RATIONALE 3](#_Toc137475444)

[1.1. General Introduction 3](#_Toc137475445)

[1.2. Rationale and justification for the Study 4](#_Toc137475446)

[a. Rationale for the Study Purpose 4](#_Toc137475447)

[b. Rationale for Doses Selected 4](#_Toc137475448)

[c. Rationale for Study Population 4](#_Toc137475449)

[d. Rationale for Study Design 4](#_Toc137475450)

[2. HYPOTHESIS AND OBJECTIVES 5](#_Toc137475451)

[2.1. Hypothesis 5](#_Toc137475452)

[a. Potential Risks 5](#_Toc137475453)

[b. Potential Benefits 5](#_Toc137475454)

[3.1. List the number of patients to be enrolled. 5](#_Toc137475455)

[3.2. Recruitment Process 6](#_Toc137475456)

[3.3. Inclusion Criteria 6](#_Toc137475457)

[3.4. Exclusion Criteria 6](#_Toc137475458)

[3.5. Withdrawal Criteria 6](#_Toc137475459)

[3.6. Patient Replacement 7](#_Toc137475460)

[4. STUDY SCHEDULE 7](#_Toc137475461)

[5. STUDY DESIGN 7](#_Toc137475462)

[5.1. Summary of Study Design 7](#_Toc137475463)

[6. METHODS AND ASSESSMENTS 7](#_Toc137475464)

[6.1. Randomisation and Blinding 8](#_Toc137475465)

[6.2. Contraception and Pregnancy Testing 8](#_Toc137475466)

[6.3. Study Visits and Procedures 8](#_Toc137475467)

[7. TRIAL MATERIAL/DEVICE 10](#_Toc137475468)

[8. SAFETY MEASUREMENTS 10](#_Toc137475469)

[8.1. Definitions 10](#_Toc137475470)

[8.2. Collecting, Recording and Reporting of “Unanticipated Problems Involving Risk to Patients or Others” – UPIRTSO events and “SAEs” to the NHG DSRB 10](#_Toc137475471)

[8.3. Safety Monitoring Plan 11](#_Toc137475472)

[8.4. Complaint Handling 11](#_Toc137475473)

[9. DATA ANALYSIS 12](#_Toc137475474)

[9.1. Data Quality Assurance 12](#_Toc137475475)

[9.2. Data Entry and Storage 12](#_Toc137475476)

[10. SAMPLE SIZE AND STATISTICAL METHODS 12](#_Toc137475477)

[10.1. Determination of Sample Size 12](#_Toc137475478)

[10.2. Statistical and Analytical Plans 12](#_Toc137475479)

[10.3. Cost-effectiveness analysis 13](#_Toc137475480)

[11. ETHICAL CONSIDERATIONS 14](#_Toc137475481)

[11.1. Informed Consent 14](#_Toc137475482)

[11.2. IRB review 14](#_Toc137475483)

[11.3. Confidentiality of Data and Patient Records 15](#_Toc137475484)

[12. PUBLICATIONS 15](#_Toc137475485)

[13. RETENTION OF TRIAL DOCUMENTS 15](#_Toc137475486)

[14. REFERENCE 15](#_Toc137475487)

**STUDY PROTOCOL**

| **BACKGROUND AND RATIONALE** |
| --- |
| General Introduction |
| Cardiovascular diseases (CVD) are the leading cause of death worldwide, accounting for 32% of global deaths [1] A staggering 38% of 17 million premature deaths caused by non-communicable diseases were attributed to CVDs [1] Hyperlipidaemia, or high low-density lipoprotein cholesterol (LDL-C), is a major risk factor for CVD leading to acute myocardial infarctions (AMI) [2] According to the National Health Survey 2019-2020, the prevalence of high blood cholesterol among Singaporeans increased from 35.5% in 2017 to 39.1% in 2019-2020.  A combination of lifestyle modifications and lipid-lowering therapy is typically recommended for individuals with high LDL-C levels to reduce the risk of CVD [3]. The World Health Organization (WHO) defines adherence as "*the extent to which the person's behaviour (including medication-taking) corresponds with agreed recommendations from a healthcare provider*" [4].  When it comes to lowering lipid levels, taking medicine as directed by the physician is the most effective way (up to 80%) compared to other modes of management such as diet, exercise and weight loss (10-20%) [5]. Unfortunately, a recent study in Singapore reported that only ~40% of patients demonstrated good adherence to their medication regimen [6,7] which is lower than the average adherence rates of 50% in developed countries [4] There are many reasons why patients are non-adherent to lipid-lowering agents. Some attributable reasons include poor understanding of the importance of medication adherence or the potential consequences of not taking their medications as prescribed, perceived or actual unpleasant side effects, forgetfulness and even cultural beliefs.  Specific to statins, addressing the nocebo effect which was well characterised in the SAMSON randomised controlled trial, could lead to 50% of individuals previously adverse to taking statins start to restart taking statins. This forms substantial basis to study nonadherence to statins, and, in particular, a deeper look into addressing the complex root causes like beliefs, values and cultural influences. This can be done through a clever combination of empathetic conversations between health coaches and the timely yet appropriate provision of information through artificial intelligence curated material, customised to a patients’ psychosocial make-up. When tethered to the biological aspect of care delivered by clinicians, the biopsychosocial triad of delivery of person-centred care first enunciated by George Engel in 1977 is complete. |
| Rationale and justification for the Study |
| Rationale for the Study Purpose |
| 1. The incidence of AMI in Singapore is expected to rise by 194.4% (482 to 1418 per 100,000 population) from 2025 to 2050. Individuals aged 40–64 years will observe the largest increase in AMI incidence (114.0%), followed by those aged 15–39 years (93.2%) and those aged ≥65 years old (28.3%) [8]. 2. Poor medication adherence portends poorer health outcomes. In Singapore, around 60% of adults not taking their medications as prescribed (as above) and this creates a considerable economic and clinical burden to individuals and health systems. 3. The use of digital technology in medication adherence has continued to grow as more healthcare providers and patients recognise its benefits in improving adherence and overall health outcome [9,10]. Digital interventions have effectively helped patients manage their medication by reminding patients to take their medications on time and providing them with more information about their medications and treatment plan [11]. In the busy world today, the provision of appropriately timed and that perceived to be important would be key to effectively convince intentionally non-adherent patients to take their medicines as prescribed. |
| Rationale for Doses Selected |
| Dosing of lipid-lowering medications adhere to local and international guidelines where relevant. |
| Rationale for Study Population |
| Hyperlipidemia remains as one of the three leading metabolic risk factors underlying AMI onset by 2050 [8]. In recent study 3 Asian ethnicities with AMI, the incidence of hyperlipidemia is projected to increase by 205% (341 to 1041 per 100,000 population) from 2025 to 2050. Locally, the high poor-adherence rates form an important basis for more to be done to address root causes. |
| Rationale for Study Design |
| This study is a multicentre, open-label, two-arm parallel randomized controlled trial. We intent to randomly assign patients with hyperlipidaemia into one of the two groups: human coach-supported Digital/AI Personal Health Assistant app (intervention group) and standard care (control group) with a 1:1 allocation ratio. |
|  |
| **HYPOTHESIS AND OBJECTIVES** |
| Hypothesis |
| We hypothesize that the use of a human coach-supported digital/AI personal health assistant (app) will improve adherence to cholesterol-lowering medications (statins with or without ezetimibe) among patients with hyperlipidaemia and suboptimal LDL-C control, when compared to standard care. |
| - 1. **Primary Objectives** |
| To evaluate the effectiveness of the human coach-supported digital/AI personal health assistant (intervention) on adherence to lipid-lowering medication at 6 and 12 months compared with those receiving standard care. |
| - 1. **Secondary Objectives**  1. To evaluate the impact of human coach-supported digital/AI personal health assistant on blood LDL-cholesterol levels at 6 and 12 months compared with those receiving standard care. 2. To evaluate the impact of human coach-supported digital/AI personal health assistant on atherosclerotic cardiovascular disease (ASCVD) risk at 6 and 12 months. 3. To evaluate the impact of human coach-supported digital/AI personal health assistant on health motivation and attitudes, self-care efficacy, self-care behaviours, and quality of life at 6 and 12 months. 4. To evaluate the acceptability of a new model of care 5. To evaluate the safety of a new model of care 6. To evaluate the (cost) effectiveness of human coach-supported digital/AI personal health assistant (intervention) on adherence to lipid-lowering medication at 12 months compared with those receiving standard care. |
| - 1. **Potential Risks and benefits:** |
| Potential Risks |
| There is minimal risk of loss of privacy breach associated with the use of mobile app. For example, if there is a data breach, someone who is not authorised could get information collected in the app. This chance is small, but it is not zero. There will be measures in place to minimum the risks in this study. |
| Potential Benefits |
| Patients in the intervention group will get full access the app and personalized Health Coaching sessions with a trained personnel to optimize management of their health condition for 12 months. |
| 1. **STUDY POPULATION** |
| List the number of patients to be enrolled. |
| This study will recruit patients with hyperlipidaemia from National University Polyclinics (NUP) and National Healthcare Group Polyclinics (NHGP). These patients must be deemed to be non-adherent to statins (DOSE score ≥ 1) and have LDL-C level above the recommended target levels (Appendix A. Clinical Guidance for Lipid Management). A total of 376 patients, with 188 patients per arm, will be recruited. |
| Recruitment Process |
| Patients will be recruited by research coordinators positioned in the polyclinics. Primary care team will screen for non adherence to statin using DOSE questionnaire, and if positive and primary care team will verbally explain the study to eligible patients. Should the patient be keen to participate in the study, the primary care provider will refer the patient to the CRC. The CRCs will confirm eligibility according to the inclusion and exclusion criteria. Informed consent will be taken by the polyclinic CRCs after routine clinic follow up and before performance of any study-related procedures (e.g downloading app, administer onboarding questionnaire, Psychometric Battery etc). Patients will then be randomly assigned to the intervention or control group. A centralised team who are not involved in the recruitment or assessment of patients will keep custody of randomization lists. |
| Inclusion Criteria |
| 1. Between 21 to 84 years old 2. Prescribed statins with or without ezetimibe for hyperlipidaemia. 3. LDL-Cholesterol above recommended target level, stratified by risk category (refer to Appendix A) 4. Medication non-adherence as defined by the "Extent to Non-adherence" sub-scale of the DOSE Non-Adherence Measure), with a score > 1 (range from 0-15) 5. Singapore residents (citizens, permanent residents, or long-term pass holders). 6. In possession of a smartphone or tablet with Android or iOS operating systems. 7. Have internet access on their mobile devices. |
| Exclusion Criteria |
| 1. Does not read or understand English. 2. Current use of smartphone medication adherence app(s) that include statins. 3. Concurrent use of PCSK9 Inhibitors in addition to statins and/or ezetimibe 4. Participation in another study that uses medications that could affect lipid levels 5. History of established atherosclerotic CVD, including but not limited to, atherosclerotic disease of coronary or carotid vessels or peripheral artery disease, documented ischaemic stroke (e.g., heart attack, cerebrovascular disease, peripheral artery disease) 6. Severe renal impairment defined as chronic kidney disease stage 4 and above. 7. Severe liver disease (Child-Pugh Class C) 8. Existing muscular-related complaints or diagnoses which may confound adverse event reporting 9. Uncorrected thyroid conditions, especially poorly-controlled hypothyroidism 10. Documented psychiatric diagnosis or history of mental illness or deemed as unable to give informed consent. 11. Currently pregnant, breastfeeding or expecting to get pregnant during the course of the study (1 year). 12. Guarded prognosis with expectant mortality within 12 months or less. |
| Withdrawal Criteria |
| Patients have the right to withdraw from (i.e., discontinue participation in) research at any time. They may be withdrawn from the study at the discretion of the investigator due if they fail to follow study procedure. |
| Patient Replacement |
| Patients who drop out may be replaced if the overall “lost to follow-up rate” is more than 20%. |
|  |
| **STUDY SCHEDULE** |
| **Table 1: Summary of Study Schedule**   \|  \| **Visit 1** \| **Visit 2** \| **Visit 3** \| **Visit 4** \| \| --- \| --- \| --- \| --- \| --- \| \|  \| **0m** \| **3m (± 2 weeks)** \| **6m (± 2 weeks)** \| **12m(± 2 weeks)** \| \| **Informed consent** \| **I, C** \|  \|  \|  \| \| **Collect demographic information from electronic medical record** \| **I, C** \|  \|  \|  \| \| **Download and install app** \| **I** \|  \|  \|  \| \| **Collect clinical data from electronic medical record** \| **I, C** \| **I, C** \| **I, C** \| **I, C** \| \| **Anthropometric measurements: Blood Pressure, height, weight, waist circumference** \| **I, C** \|  \| **I, C** \|  \| \| **Pill counts** \|  \| **I, C** \| **I, C** \| **I, C** \| \| **Questionnaires** \| \| \| \| \| \| - **Onboarding questionnaire** \| **I, C** \|  \|  \|  \| \| - **Dose Non-Adherence Measure** \| **I, C** \| **I, C** \| **I, C** \| **I, C** \| \| - **Psychometric Battery Questionnaires** \| **I, C** \| **I, C** \| **I, C** \| **I, C** \| \| **Clinical Pharmacist follow-up/review** \|  \| **I, C** \| **I, C** \| **I, C** \| \| **Human-AI-health coaching** \|  \| **I** \| **I** \| **I** \|   I = intervention, C = control |
| STUDY DESIGN |
| Summary of Study Design |
| This study is a multicentre, randomized controlled trial (RCT) on 376 hyperlipidaemia patients who are nonadherent to statins, with suboptimal LDL-C levels. These patients will be randomly assigned into one of the two groups: intervention group or standard care (control group) with a 1:1 allocation ratio. The intervention group will receive personalised feedback through the app coupled with human coaching on top of usual clinical care for cholesterol management. The control group will receive usual standard of care for lipid management but will not receive the personalised app nor have access to health coaching. |
| **METHODS AND ASSESSMENTS** |
| 1. **Clinical data:** Clinical data will be collected on a patient’s demographic details, past medical history, medication use, blood test results and anthropometric measurements. 2. **Questionnaires**: Onboarding questionnaire, DOSE Non-Adherence Measure and Psychometric Battery Questionnaires will be administered by the CRC 3. **Pill count**: Patients are required to bring along their prescribed lipid-lowering medications during their follow-up visits (@3m, 6m, and 12m). CRC will carry out pill counts with the patients during the visits. 4. **Medical care follow-up**: Standard-of-Care clinical pharmacist will follow-up with patients, titrating lipid-lowering medication (such as statin, ezetimibe etc) as required, and review and take action clinical blood test results (Appendix B; e.g lipid level, blood creatinine kinase and liver function tests where relevant). 5. **Human-AI-health coaching (For intervention only):** Health coach will use the information gathered by the AI chatbot to guide the targeted behavioural intervention during phone consultation. The scope of coaching will be strictly related to the medication adherence and general well-being. The coach will not start, stop, or titrate any medication. Coach will escalate concerns to clinical pharmacists when deemed fit. 6. **Focus group discussion (sub-study):** This will be conducted with a nested sample of 30-50 intervention group patients. The aims are: (a) to collect insights from intervention patients on their experiences with the app and human health coaching, (b) insights into which intervention components work best for them and under what circumstances, (c) insights into concerns which might impact intervention effectiveness, (d) factors that draw their participation and sustained engagement, (e) factors that deter them from sustainable engagement, (f) factors that may lead other CVD patients to be more inclined to partake in such a intervention and (g) ideas and suggestions to make the intervention more appealing and effective. |
|  |
| Randomisation and Blinding |
| DOSE-Non-adherence score 1 or higher consenting patients who have completed baseline assessment will be randomly assigned into the intervention group (app) or the control group (standard care). The CRC will use the Study ID list to perform randomization and randomly allocate patients via block randomisation method with random permuted block sizes of 4 and 6. The randomisation will be computed using the R statistical software package by a statistician who will not be involved in the recruitment process. The random block sizes will be used to allocate patients to the intervention and control groups at a 1:1 ratio. Study allocation based on assigned Study ID will only be made known to the patients from the attending CRC after eligibility and consent are sought. |
| Contraception and Pregnancy Testing |
| The use of contraception will not be considered in this study. Pregnant and breastfeeding women will be excluded from this study. |
| Study Visits and Procedures |
| **Pre-screening, recruitment and Visit 1 (month 0)**  Primary care team will screen for non-adherence to statin using DOSE questionnaire, and if positive and primary care team will verbally explain the study to eligible patients. Should the patient be keen to participate in the study, the primary care provider will refer the patient to the CRC.  Upon recruitment and after randomisation, patients will be informed if they are allocated to be in the intervention or control group. Polyclinic CRCs will email a centralized team who are not involved in the screening and assessment of patients for the randomization outcome. Only the centralized team will have access to the randomization list that was pre-generated. The polyclinic CRCs will be blinded until the randomization outcome upon being informed.  CRC will administer the remaining questionnaires and take anthropometric measurements. Lastly, CRC will collect demographic and clinical information from electronic medical record.  **INTERVENTION:** Patients will be required to install the intervention app. The CRC will take the patient through the app features and answer any questions they may have.  Patients will receive curated, reliable and up-to-date medical information in an easily digestible and understandable format via in-app text message. The app can also send reminders via in-app text messages or push notifications to remind patients to take their medicine and their appointment dates. Our Artificial intelligence (AI)–based chatbots will prompt patients to engage in conversation to uncover their barriers to medication adherence and assist with setting personal goals.  **Follow-up Visits (Visit 2 @Month 3, Visit 3 @ Month 6 and Visit 4 @ Month 12**  The CRC will remind patients to bring their lipid-lowering medication before the study visits.  During the visit, the CRC will carry out pill counts, administer questionnaires and collect clinical information from electronic medical record. Anthropometric measurements will be taken at Visit 3 (month 6) only.  Clinical pharmacist will review clinical blood test results, monitor for safety and titrate lipid-lowering medications as required.  **Intervention Group:** Health coach will use the information gathered by the AI-based chatbot, such as patient's readiness to change, perceived barriers and misbeliefs to guide the targeted behavioural intervention via in-app text message or phone consultations. The scope of coaching will be strictly related to the medication adherence and general well-being. The coach will not start, stop, or titrate any medication.  **Focus group discussion (sub-study):** 30-50 Intervention patients who give consent for this substudy will be contacted by study team. The discussion will be held either in-person or virtually and will take approximately 3-4 hours. |
| **Discontinuation Visit and Procedures** |
| In the case of patients withdrawing, or being terminated from the study due to unforeseen circumstances prior to the 1-year mark, they will be required to sign the Acknowledgment of Withdrawal form, and the CRC will have to acknowledge on the same form as well as confirmation. Patients’ decision to withdraw will not affect their medical care or any benefits to which they are entitled.  The CRC will access patient’s electronic medical record to collect clinical information (such as medications, blood test results, clinical events etc).  In cases where patient is uncontactable (“lost to follow-up”), every possible effort must be made to contact the patient and determine the reason for discontinuation or withdraws. Reasons for discontinuation or withdraws should be recorded, if possible. Clinical data collected prior to withdrawal will be retained for analysis, unless the patient disapproves it. |
| **TRIAL MATERIAL/DEVICE** N/A |
| **SAFETY MEASUREMENTS** |
| Definitions |
| **Adverse Event** – Any untoward or unfavourable medical occurrence in a patient or clinical investigation patient administered with a pharmaceutical product and which does not necessarily have a causal relationship with this treatment.  **UPIRTSO event** –refers to problems, in general, to include any incident, experience, or outcome (including adverse events) that meets **ALL** of the following criteria:   1. **Unexpected**   In terms of nature, severity or frequency of the problem as described in the study documentation (eg: Protocol, Consent documents etc).   1. **Related or possibly related to participation in the research**   Possibly related means there is a reasonable possibility that the problem may have been caused by the procedures involved in the research; and   1. **Risk of harm**   Suggests that the research places patients or others at a greater risk of harm (including physical, psychological, economic, or social harm) than was previously known or recognized.  **Serious Adverse Event** – A serious adverse event or reaction is any untoward medical occurrence which:   1. Results in or contributes to death; 2. Is life-threatening; 3. Requires inpatient hospitalisation or prolongation of existing hospitalisation; 4. Results in or contributes to persistent or significant disability or incapacity; or 5. Results in or contributes to a congenital anomaly or birth defect. |
| Collecting, Recording and Reporting of “Unanticipated Problems Involving Risk to Patients or Others” – UPIRTSO events and “SAEs” to the NHG DSRB |
| The PI is responsible for the accurate documentation, investigation, follow-up and timely reporting of all UPIRTSOs and SAEs. Only related SAEs (definitely/ probably/ possibly) will be reported to DSRB. “Related” means there is a reasonable possibility that the event may have been caused by participation in the clinical trial  **Assessment of Events**  The PI must make a judgment about the **expectedness**, of an event. If the event is an adverse event, the PI must make a judgment about the **causality** of the adverse event. The PI must also analyse the event and state whether protocol / consent form revisions are required.  In the event of UPIRTSOs/Expected SAEs, the PI is responsible to ensure that adequate medical care is provided to the patient for treatment of adverse events.  **Reporting Timeline for UPIRTSO Events to the NHG DSRB.**  The PI must report all UPIRTSOs that occur during the conduct of a research project to the DSRB, in accordance with the timelines set by DSRB.   1. **Urgent Reporting:** All problems involving local deaths, whether related or not, should be reported immediately – within 24 hours after first knowledge by the PI. 2. **Expedited Reporting**: All other problems must be reported as soon as possible but not later than 7 calendar days after first knowledge by the PI. |
| Safety Monitoring Plan |
| Safety and wellbeing will be assessed continuously in all patients participating in the study throughout the trial period. PI and co-investigators (co-Is) will provide day-to-day oversight of the trial. They will assure that informed consent is obtained prior to performing any research procedures, that all patients meet eligibility criteria and that the study is conducted according to the DSRB-approved research plan.  PI will evaluate the progress of the research study, including recruitment and retention, protocol deviations and an assessment of the timeliness and quality of the data on a biannual basis. They will also review the collected data including AEs and SAEs, unanticipated problems and withdrawal to determine if there is any change to the anticipated benefit-to-risk assessment of study participation. PI will ensure all protocol deviations, AEs and SAEs are reported according to the applicable regulatory requirements.  In addition to Serious Adverse Events that are unexpected or related, all Expected SAEs also should also be reported as soon as possible but not later than 7 calendar days after first knowledge by the investigator, and any additional relevant information about the events should be reported within 8 calendar days of making the initial report.  All research data will be stored within the institution. The completed questionnaires and informed consent forms will be kept in a locked cabinet in the respective recruitment sites. Only authorized study staff (PI, Co-I and CRCs) will have access to the research data. This is to ensure that all study information remains safe and secure. |
| Complaint Handling |
| Patients can contact the CRCs when they have complaints. The initial recipient of a complaint should communicate to PI without delay. The PI should acknowledge receipt as soon as possible, make every effort to resolve the issue after preliminary investigation. Any communication with the complainant should be documented. |
| **DATA ANALYSIS** |
| Data Quality Assurance |
| To ensure the accuracy and reliability of the data, PI, co-Is, CRCs, pharmacists, health coaches must be familiar with the protocol and all study-specific procedures. The app used has passed usability testing and load testing prior to being introduced to the study. This ensures that the data collected is uniform throughout all the patients in the intervention group. |
| Data Entry and Storage |
| It is important to store the data securely and used in a way its integrity is not compromised. Data obtained from the app will be stored on a local secured cloud platform and only accessible to authorised study team members (PI, co-Is, CRCs, pharmacists). Clinical data will be stored in a secure database, accessible only to the PI, co-Is, CRCs, pharmacists. The use of the data from the study will be controlled by the PI. |
| **SAMPLE SIZE AND STATISTICAL METHODS** |
| Determination of Sample Size |
| A total of 376 patients (188 per arm) are to be recruited. The computation takes into account a) 5 level of significance, b) desired statistical power of at least 90%, c) the potential application of a mixed-effect model in view of the multi-centre design, and to cater for the random effects of repeated measurements, d) a small effect size to be detected (i.e., <0.2), e) fewer than 10 predictors to be accommodated in model building and f) no more than 20% drop outs. A post-hoc power calculation should be facilitated after the data analysis is performed. |
| Statistical and Analytical Plans |
| The data collected will be screened for accuracy, missing data, outliers, and statistical assumptions. Following the principles of intention-to-treat (ITT) analysis, individuals will be retained in the group they were randomized to. At each measurement time, efforts will be made to minimize missing outcome data, and the reasons for individuals' loss of follow-up will be recorded. Missing data will be imputed using averages of answered items with no more than 20% missing data. When more than 20% of the items are missing, the overall score is not computed, and the data is considered missing. Sensitivity analyses will also be performed to determine the robustness of the assumption about missing data.  Descriptive statistics of baseline and follow-up data will be reported by mean and standard deviation for normally distributed variables, median and interquartile range for non-normally distributed variables, and proportions (count and percentage) for categorical variables. Continuous variables that are not normally distributed will be approximately normalised by using transformations and categorisations whenever applicable. Between group comparisons (by gender, age group, years since diagnosis, etc) will be made using Pearson χ2 or Fisher's exact test for categorical data, whereas continuous data will be tested with independent t-test and Mann–Whitney U test to evaluate changes between baseline and end-of-trial measures.  This study will also include regression/ modelling analyses to adjust potential confounders in this study such as gender, disease duration, number of comorbidities, and hypercholesterolemia treatment modalities (statins only, or ezetimibe, or statins plus ezetimibe). Subgroup analysis may be performed if the potential confounders found to be significant.  STATA (StataCorp Statistics for Windows, Version X, College Station, Texas) and R version X (The R Foundation for Statistical Computing; Vienna, Austria) will be used for all statistical analysis. All statistical tests will be performed using two-sided tests at the 0.05 significance level. |
| Cost-effectiveness analysis Economic evaluation: A cost-effectiveness study will be conducted to produce evidence about the long-term economic benefits of the app. A decision analytic model will be constructed to estimate the value for money of implementing the app in Singapore. The structure of the model will be defined by the natural history of a patient with hypercholesterolaemia. The analysis will consider only the health system perspective. The following three parameters are needed for the decision analytic model: transition probabilities, costs, and effectiveness measures |
| 1. ***Transition probabilities:*** The transition probabilities will be sourced from the data collected in the trial (e.g., risk categories, adherence rate), and from the literature. 2. **Costs:** Intervention cost will be estimated using the information available within the trial budget. The cost of developing the app as a one-off cost, training the personnel, and maintenance cost will be calculated. Furthermore, the costs of statin treatment and the costs of cardiovascular events will be estimated and will be used as model inputs. Cost-effectiveness, healthcare utilisation, and direct medical costs will be collected. 3. **Effectiveness measure:** The study will use several effectiveness measures. For health economic evaluations, effectiveness measures must be either in natural measurements, such as hospital avoidance, or patient-reported outcome measures, such as utility estimates. The effectiveness measures that we would use are EQ-5D-5L utility estimates (quality-adjusted life years - QALY), the number of hospital admissions, ED presentations and unplanned primary care visits avoided. |
| - 1. **Focus Group Data Analysis**   All focus group data will be analyzed using Framework Analysis [12]. This involves both inductive and deductive approaches to critically examine the data and generate conceptual themes and theme-categories that illuminate programme feasibility and acceptability. The focus will be on developing an evidence-based framework for informing intervention enhancement for large scale implementation. Framework analysis is a valuable tool in applied health policy research with specific questions, a purposive sample, a priori issue, and aims to meet specific information needs to generate outcomes and recommendations. It involves several steps of (a) multiple readings and line-by-line coding to delineate the central concepts that emerged from the interviews; (b) forming themes and sub-themes by combining codes that are similar; and (c) axial coding to develop and refine possible theme-categories. Appointed members of the research team will review and delineate emergent themes and sub-themes in their analysis, and thereafter present to each other for discussion and confirmation of preliminary findings. This will be followed by presentation of preliminary findings by the larger research team during periodic meetings for further discussion and revision. In the final stage, all major categories, themes and sub-themes will be named, defined, operationalized related to one another and mapped with supporting quotes from interview transcripts, thereby generating an overall theoretical framework. Research rigor and trustworthiness will be ensured by adopting strategies such as prolonged engagement with the data, peer debriefing, thick descriptions of data, negative case analysis, members checking, as well as triangulation of data, investigator, and theory. The QSR NVIVO software package will be used to manage the data. |
| **ETHICAL CONSIDERATIONS** |
| Informed Consent |
| Prior to participation in the study, each patient must give written informed consent according to ICH / GCP and to the regulatory and legal requirements of the participating country. The consent form must be signed before performance of any study-related activity.  Before entry into the study, PI/co-Is or CRC must explain the nature of the study, including aims, methods, anticipated benefits, potential risks of the study and any other possible discomfort it may entails, to potential patients. Patients will be informed that their participation is voluntary and that they may withdraw consent participation at any time. Furthermore, patients will be informed that withdrawal from participation will not affect the care that he/she will receive. Finally, they will be told that the investigators may access their medical records after the study end for long-term follow-up (for up to 20 years) without violating the confidentiality of the patients and to the extent permitted by local law(s) and regulations. The patients will be given enough time to read the informed consent form and the opportunity to ask questions.  Each signature must be personally dated by each signatory, and the ICF and any additional patient-information form retained by the Investigator as part of the trial records. A signed copy of the informed consent and any additional patient information must be given to each patient. The process of obtaining informed consent should be documented in the patient source documents. |
| IRB review |
| This study will be conducted in accordance with the ethical principles that have their origin in the Declaration of Helsinki and that are consistent with the Good Clinical Practice and the applicable regulatory requirements.  The study protocol, including the final version of the Informed Consent Form, must be approved in writing by the NHG Domain Specific Review Board (DSRB), prior to enrolment of patient into the study. The principal investigator is responsible for informing the DSRB of any amendments to the protocol or other study-related documents, as per local requirement.  Any necessary extensions or renewals of IRB/IEC approval must be obtained for changes to the study such as amendments to the protocol, the ICF or other study documentation before implementation.  The Investigator will report promptly to the IRB/IEC any new information that may adversely affect the safety of the patients or the conduct of the study. The Investigator will submit written summaries of the study status to the IRB/IEC as required. On completion of the study, the IRB/IEC will be notified that the study has ended. |
| Confidentiality of Data and Patient Records |
| All information obtained during the study will be regarded as confidential. No data will be disclosed to any third party without obtaining permission from the patients, except for the purposes of monitoring, auditing, or inspection by the Regulatory Authorities and Ethic Committees. The use of the data from the study will be controlled by the principal investigator.  PI must ensure confidentiality and compliance with applicable data privacy protection laws and regulation. Each patient will be identified by either screening or patient ID number only. All source document and essential documents shall be stored in the locked cabinet. Electronic data shall be protected with password and stored in a secured computer. Only the investigators or designated analysts/personnel may access the data. |
|  |
| **PUBLICATIONS** |
| The outcome of this study will be presented at national and international meetings and published in peer-reviewed journals. We would consider authorship to include all of the following: 1) conception and design or analysis and interpretation of data, or both; 2) drafting of the manuscript or revising it critically for important intellectual content; and 3) final approval of the manuscript submitted. Participation solely in the collection of data does not justify authorship but may be appropriately acknowledged in the Acknowledgment section. |
|  |
| **RETENTION OF TRIAL DOCUMENTS** |
| Records for all patients, including CRFs, all source documentation (containing evidence to study eligibility, history and physical findings, laboratory data, results of consultations, etc.) as well as IRB records and other regulatory documentation will be retained by the institute PI in a secure storage facility as per ICH GCP and applicable regulatory requirement(s). These records will be retained for the period required by the institution policy. |
| **REFERENCE** |
| [1] World Health Organization. World Health Organisation. Fact sheets on cardiovascular diseases (CVDs). 2021. <https://www.who.int/en/news-room/fact-sheets/detail/cardiovascular-diseases-(cvds)>.  [2] Liberopoulos EN, Florentin M, Mikhailidis DP, Elisaf MS. Compliance with lipid-lowering therapy and its impact on cardiovascular morbidity and mortality. Expert Opin Drug Saf 2008; 7: 717–25.  [3] Michos ED, McEvoy JW, Blumenthal RS. Lipid Management for the Prevention of Atherosclerotic Cardiovascular Disease. *N Engl J Med* 2019; **381**: 1557–67.  [4] World Health Organization. Adherence to long-term therapies : evidence for action. 2003. <https://apps.who.int/iris/handle/10665/42682>.  [5] Singapore Heart Foundation. Statins: The Most Misunderstood Lifesaving Drug. [Statins: The Most Misunderstood Lifesaving Drug \| Singapore Heart Foundation (myheart.org.sg)](https://www.myheart.org.sg/heart-news/statins-the-most-misunderstood-lifesaving-drug/). [Accessed on 9 June 2023]  [6] Chew SM, Lee JH, Lim SF, Liew MJ, Xu Y, Towle RM. Prevalence and predictors of medication nonadherence among older community‐dwelling people with chronic disease in Singapore. *Journal of Advanced Nursing* 2021; **77**: 4069–80.  [7] Lee CS, Tan JHM, Sankari U, Koh YLE, Tan NC. Assessing oral medication adherence among patients with type 2 diabetes mellitus treated with polytherapy in a developed Asian community: a cross-sectional study. BMJ Open. 2017 Sep 14;7(9):e016317.  [8] Nicholas W.S. Chew, et. al., Trends and predictions of metabolic risk factors for acute myocardial infarction: findings from a multiethnic nationwide cohort. The Lancet Regional Health - Western Pacific. 2023. <https://doi.org/10.1016/j.lanwpc.2023.100803>.  [9] Li A, Del Olmo MG, Fong M, Sim K, Lymer SJ, Cunich M, Caterson I. Effect of a smartphone application (Perx) on medication adherence and clinical outcomes: a 12-month randomised controlled trial. BMJ Open. 2021 Aug 9;11(8):e047041.  [10] Peng Y, Wang H, Fang Q, Xie L, Shu L, Sun W, Liu Q. Effectiveness of Mobile Applications on Medication Adherence in Adults with Chronic Diseases: A Systematic Review and Meta-Analysis. J Manag Care Spec Pharm. 2020 Apr;26(4):550-561.  [11] Márquez Fosser S, Mahmoud N, Habib B, Weir DL, Chan F, El Halabieh R, Vachon J, Thakur M, Tran T, Bustillo M, Beauchamp C, Bonnici A, Buckeridge DL, Tamblyn R. Smart about medications (SAM): a digital solution to enhance medication management following hospital discharge. JAMIA Open. 2021 Jun 18;4(2):ooab037. doi: 10.1093/jamiaopen/ooab037. PMID: 34159299; PMCID: PMC8211568.  [12] Gale, N. K., Heath, G., Cameron, E., Rashid, S., & Redwood, S. (2013). Using the framework method for the analysis of qualitative data in multi-disciplinary health research. BMC Medical Research Methodology 13,117 https://doi.org/10.1186/1471-2288-13-117 |
